# Detection of high frequency of MAD20 allelic variants of *Plasmodium falciparum* merozoite surface protein 1 gene from Adama and its surroundings, Oromia, Ethiopia

**DOI:** 10.1186/s12936-021-03914-9

**Published:** 2021-09-27

**Authors:** Temesgen File, Tsegaye Chekol, Gezahegn Solomon, Hunduma Dinka, Lemu Golassa

**Affiliations:** 1 Department of Applied Biology, Adama Science and Technology University, P.O.Box 1888, Adama, Ethiopia; 2 Aklilu Lemma Institute of Pathobiology, Addis Ababa University, P.O.Box 1176, Addis Ababa, Ethiopia

**Keywords:** Genetic polymorphism, Msp-1, Multiplicity of infection, *P. falciparum*

## Abstract

**Background:**

One of the major challenges in developing an effective vaccine against asexual stages of *Plasmodium falciparum* is genetic polymorphism within parasite population. Understanding the genetic polymorphism like block 2 region of *merozoite surface protein-1* (*msp-1*) gene of *P. falciparum* enlighten mechanisms underlining disease pathology, identification of the parasite clone profile from the isolates, transmission intensity and potential deficiencies of the ongoing malaria control and elimination efforts in the locality. Detailed understanding of local genetic polymorphism is an input to pave the way for better management, control and elimination of malaria. The aim of this study was to detect the most frequent allelic variant of the *msp-1* gene of *P. falciparum* clinical isolates from selected health facilities in Adama town and its surroundings, Oromia, Ethiopia.

**Methods:**

One hundred thirty-nine clinical isolates were successfully amplified for *msp-1* gene using specific primers. Nested PCR amplification was conducted targeting K1, MAD20, and R033 alleles followed by gel electrophoresis for fragment analysis. Based on the detection of a PCR fragment, infections were classified as monoclonal or multiple infections.

**Results:**

19 different size polymorphism of *msp-1* gene were identified in the study, with 67(48%) MAD20, 18 (13%) K-1 and 18 (13%) RO33 allelic family. Whereas, the multiple infections were 21(15%), 8 (5.8%), 4(2.9%), 3(2.2%) for MAD20 + K-1, MAD20 + RO33, K-1 + RO33, and MAD20 + K-1, RO33, respectively. The overall Multiplicity of infection (MOI) was 1.3 and the expected heterozygosity (He) was 0.39 indicating slightly low falciparum malaria transmission.

**Conclusion:**

The status of *msp-1* allele size polymorphism, MOI and *He* observed in the study revealed the presence of slightly low genetic diversity of *P. falciparum* clinical isolates. However, highly frequent MAD20 allelic variant was detected from clinical isolates in the study area. Moreover, the driving force that led to high predominance of MAD20 allelic variant revealed in such malaria declining region demands further research.

**Supplementary Information:**

The online version contains supplementary material available at 10.1186/s12936-021-03914-9.

## Background

Despite an enormous effort to control and eventually eliminate malaria, studies reveal that it is still a major public health problem, especially in sub- Saharan Africa (SSA) where more than 90% of the disease burden prevails [1, 2]. About 68% of Ethiopian population inhabits in 75% of the countries land mass that is malarious, where *Plasmodium falciparum* and *Plasmodium vivax* accounts for 70% and 30%, respectively [3]. Studies revealed that, multiple factors greatly affected malaria control and elimination efforts. From which the frequent emergence and spread of genetic diversity of *P. falciparum* is prominent. High genetic diversity is not only an indicator of its evolutionary success [4] but also, the intensity of transmission [5] that pose potential challenges in malaria control programmes [6].

Molecular characterization of *P. falciparum* enables us to investigate the genetic diversity of infection with consideration of various factors, such as disease phenotype, age and host immunity [7]. Genetic diversity of *P. falciparum* is usually determined through genotyping of the polymorphic regions block 2 of *msp-1* [6, 8, 9]. MSP1 is involved in erythrocyte invasion and is one of the major *P. falciparum* blood-stage malaria vaccine targets [10–12]. MSP1 is a 190 KDa surface protein encoded by the *msp1* gene located on chromosome 9 and contains 17 blocks of sequences flanked by conserved regions [9, 13, 14]. The precise functional role of *msp1* during invasion has not been fully evaluated, and its macromolecular characterization is incomplete [15].

*msp-1* markers are useful to investigate genetic diversity, multiplicity of infection (MOI) and parasite carriage. Polymorphism in *msp1* and *msp2* have been frequently reported from different parts of the world. Of the 17 blocks of *msp1*, block 2 is the most polymorphic region characterized into three allelic families (K1, MDA20 and R033). Based on the variation in length and sequence diversity, this region is a commonly targeted part in determining genetic diversity and MOI in clinical isolates of *P. falciparum*.

Even though genetic diversity of *P. falciparum* has been extensively studied in different parts of the world, limited data are available from Ethiopia. The aim of this study was, to assess genetic diversity of block 2 region of *msp-1* gene of *P. falciparum* clinical isolates from three districts in central Ethiopia.

## Methods

### Study sites

**Fig. 1:**
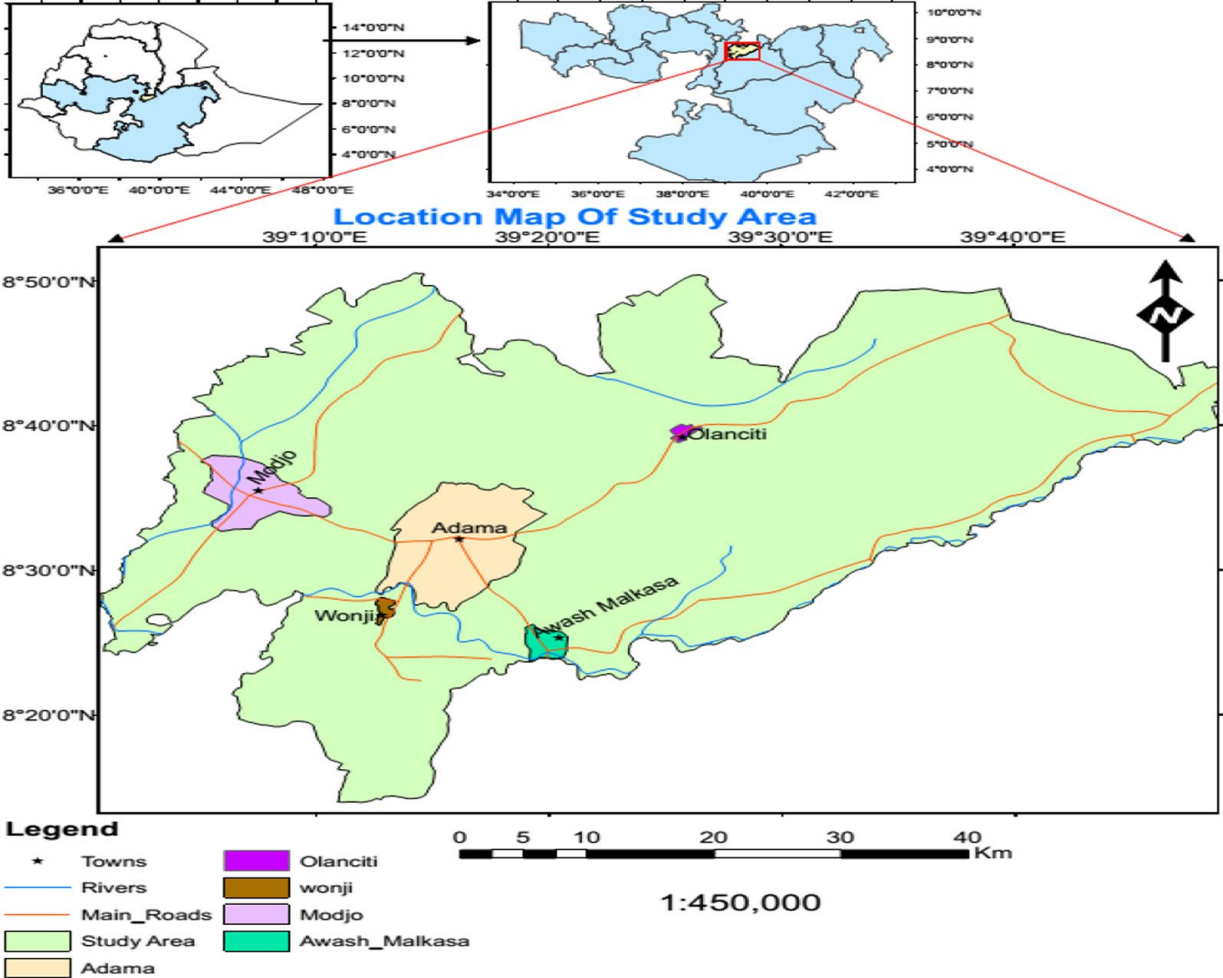
Map of the study area, showing sample collection sites (Adama, Modjo, Wonji, Awash Malkasa, and Olanciti)

Health facility based cross-sectional study conducted at Adama, Modjo, Wonji, Awash Malkasa and Olanciti towns from September 2019 to August 2020. These sample collection sites include Adama city administration, Adama district which includes Wonji and Awash Malkasa, Modjo town capital of Lume district, Olanciti the capital of Boset district. The location of these study sites is as shown in (Fig. 1). Patient data and sample collection, was performed from purposively selected health facilities at each site depending on their patient caseload, physical location and the availability of qualified and experienced medical laboratory technologist previously participated in similar research work. Adama is the major town next to the main capital in central Ethiopia. It is found at about 99 km southeast of Addis Ababa. The location of the other sites are Modjo at 16 km northwest, Wonji at 8 km south, Awash Malkasa at 15 km southeast and Olanciti at about 23 km northeast of Adama.

The study sites are located in the Rift Valley areas having rain fall pattern that is heavy from mid-June to mid-September followed by major malaria transmission season and shorter rainy period in March accounting for minor malaria transmission [16]. The catchment population of the study site estimated to reach 800,000 inhabitants.

Adama and its surrounding area is a well-known malaria endemic region in central Ethiopia. The major factors that account for such malaria endemicity are; its physical location in Rift Valley area of Ethiopia, latitudinal location which varies from1436 to 1850 m above sea level, rainfall patterns, average annual temperature that varies from 16–32 °C that is favourable for the breeding of *Anopheles arabiensis* (the predominant malaria vector in the region), and various micro ecological factors that favour mosquito breeding [17].

### Clinical data, blood sample collection and processing

One hundred thirty-nine microscopically-confirmed *P. falciparum* infected patients were included in the study. The age of study participants were ranged from 1 to 66. The inclusion criteria for the study were uncomplicated malaria patient with the history of fever onset since 24 h of the clinical examination. Blood samples were collected through finger prick for dry blood spot (DBS) preparation was held from September 2019 to August 2020 by trained medical laboratory technologists from the catchment area of those selected health facilities in the study area. After consent of the patients or guardians, the spotted blood on Whatman TM 3MM filter paper was allowed to air dry in dust free area. The DBS were placed in a zip-lock bag with silica gel temporarily stored at 0–4 °C to prevent DNA degradation. For longer time storage of DBS was kept in deep freeze (− 20 °C) at Adama regional research laboratory.

### Microscopy and parasite count

Microscopic examination was conducted for both thick and thin blood smear following the national malaria microscopy protocol. All slides examined by two independent laboratory technologists to determine species identification for *P. falciparum* and its parasite density. In case of discordance, a third laboratory technologist read the slides. In addition, parasite density was estimated by counting and recording the asexual stage of the parasite per 200 white blood cells (WBC) in thick film. Moreover, when the sexual form (gametocytes) have been seen, the slides were excluded from the count. The parasite density of the asexual stage was estimated by counting the number of WBC by field examined assuming that 8000 WBC were present in 1 µl of blood. Thus, parasite density per microlitre (μl) of blood was calculated by using the following formula [7, 18, 19]:

1 $${\text{Parasite density}}/{\upmu} {\text{l}}=\frac{\begin{array}{c} \\ {\text{Number of asexual parasite per}}200{\text{WBC}} \\ \times\, {\text{absolute or assumed WBC}}/{\upmu} {\text{l}} \end{array}}{200}$$

For comparison with ranked order variables, parasitaemia were categorized in to five levels: L1 (< 50 parasite/µl blood), L2 (50–499 parasite /µl blood), L3 (500- 4999 parasite /µl blood), L4 (5000–49,999 parasite/µl blood), and L5 (≥ 50,000 parasite/µl blood) [20].

### Extraction of the parasite DNA

Extraction of the parasite genomic DNA and genotyping the polymorphic region of *msp-1* were conducted at malaria research laboratory, Akililu Lemma Pathobiology Institute, Addis Ababa University (AAU). Genomic DNA of *P. falciparum* extracted from approximately 200 µl of frozen blood samples spotted on Whatman 3 filter paper for nested PCR amplification. 0.5% Tween® 20 (Sigma-Aldrich, USA) was used to lyse RBC; tracked by treatment with 6% chelex ® 100 (Sigma-Aldrich, USA) and heat treatment in water bath at 96 °C following the optimized standard operating procedure (SOP) to free the parasites DNA [9].

### PCR amplification for genotyping of msp-1 gene and gel electrophoresis

Nested PCR amplification targeting the unique sequence of *18 srRNA* gene was held by using specific primer pairs for molecular detection of *P. falciparum* from the isolates [21]. In the present study, the polymorphic region of the confirmed *P. falciparum msp-1* gene (block 2) was used as a genetic marker for the genotyping of parasite populations. The primers and PCR conditions used during this study were slightly modified from the previously described work [9, 22] (Additional file 1). Briefly, all reactions carried out in a final volume of 20 µl. In the first round (N1) reaction containing 0.5 µl of each primer, 5 × FIREPol®.Master Mix (MM), 11 µl of nuclease free water aliquot to 16 µl to which 4 µl of DNA template was added. In nested (N2) reaction, 2 µl of the amplicon product was used. The PCR amplification profile for both N1 and N2 reactions includes; initial denaturation at 95 °C at 3 min, denaturation at 94 °C for 1 min, annealing 58 °C for 1 min, elongation 72 °C for 2 min and final elongation at 5 min [9, 22]. The PCR reaction mixture incubated in a thermal cycler (VWR) Schmidt, Germany. To monitor the quality the protocol allele specific positive control 3D7 and DNA free negative control were included in each reaction. Separation of the PCR product was performed on 2% agarose gel electrophoresis stained with ethidium bromide [9]. Stained agarose gels visualized under Benchtop 2UV trans-illuminator (UVP) USA and photographed to estimate band size in relation to 50 bp DNA ladder (Invitrogen, by thermal Fisher-scientific) (Additional file 2). Infections considered as monoclonal when a single PCR fragment was detected on each locus and polyclonal when more than one fragment identified on a locus. Polymorphism in each allele family was analysed by assuming that one band represented one amplified DNA fragment derived from a single copy of *P. falciparum msp-1*. Multiplicity of infection (MOI) was defined as the average number of detected *P. falciparum* genotypes per infected patient. Allele for each family were considered the same when the fragment size is less than 20 bp [23].

### Data analysis

The data were analysed, after entering and processing it by using Statistical Package for Social Sciences (SPSS version 20). To examine endemicity or potential importation of the *msp-1* allelic variants, confirmed malaria patients with *P. falciparum* were categorized in those having travel history to other places where malaria is endemic and those not having travel history in the preceding 30 days. MOI for *P. falciparum* was calculated as a total number of parasite genotypes for the same gene and the number of PCR positive isolates. Descriptive analysis performed to compare the distribution of different allele families in relation to patient data.

To test the correlation of two variables, Pearson correlation test was used. Pearson Chi square test was also conducted for statistical comparison of categorical variables. P < 0.05 was used to test the level of statistical significance to accept or reject the hypothesis.

The expected heterogeneity $(He)$ was calculated by the formula;

2 $$He=\left(\frac{n}{n-1}\right)\left(1-\sum {p}^{2}\right),$$

where “n” stands for the number of the isolates analyzed and “p” represents the frequency of each different allele at a locus.

### Ethical considerations

Ethical approval of the study was obtained from Institutional Ethical Review Board of ASTU, certificate reference number RECSoANS/BIO/01/2019 and approval of Oromia Regional State Health Bureau. In addition, written informed consent obtained from parents or guardian prior to recruitment.

## Results

### Socio-demographic and parasitological data

One hundred thirty-nine samples from *P. falciparum* patients were successfully analysed for *msp-1* allelic diversity. A total of 68.3% of the study participant were males. The age of the study participants ranged from 1 to 66. Mean ± SD (27.0 ± 13.6*) years. Asexual parasite density ranged from 64 – 104,320 parasites/µl with a geometric mean of 5,654 parasites/µl. Of all study participants 83 (60%) were from urban inhabitants, and only 15 (11%) were having recent travel history to other malarious area. Of all the study subjects, by occupation 74% *P. falciparum* malaria cases were detected from students, daily labourers and farmers (Table 1).

**Table 1:** Socio-demographic characteristics and parasitological data of the study population at Adama and its surroundings (n = 139)

Patient characteristics	Value
Mean age (year)	27.0 ± 13.6 * (SD)
Age range (year)	1–66
Sex ratio (male/female)	95/44
Residence (urban/rural)	83/56
Travel history to malarious area	15 (11%)
Educational level	
Not attended formal education	18 (13%)
Attended primary school	71 (51%)
Attended secondary school and above	50 (37%)
Occupation	
Farmer	26 (19%)
Housewife	14 (10%)
Daily labourer	33 (24%)
Government employee	14 (10%)
NGO employee	2 (1.4%)
Business man	6 (4.3%)
Student	44 (32%)
Geometric mean of parasitic density (P/µl) of blood	5654.0
Parasite density range (P/µl) of blood	64–104,320.0
Parasitaemia level	
50–499 P/µl of blood	9 (6.4%)
(500–4999 P/µl of blood)	63 (45.3%)
(5000–49,999 P/µl of blood)	63 (45.3%)
(≥ 50,000 P/µl of blood)	4 (2.9%)

### Geometric mean of the parasite density across different age groups

**Fig. 2:**
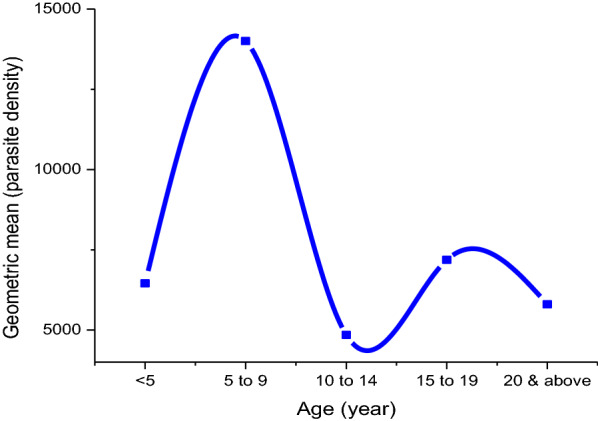
Relationship between geometric mean of the parasite density of *P. falciparum* patients with age groups in Adama and its surroundings (n = 139)

Analysis of the geometric mean of *P. falciparum* parasite density across patients of different age groups has shown that school aged children (5–14 years) carry disproportionate burden of the infection (Fig. 2). However, the correlation between parasite density with patient’s age is not statistically significant (Pearson’s correlation = 0.12, P = 0.6).

### Allele typing and diversity profile across different age groups

**Fig. 3:**
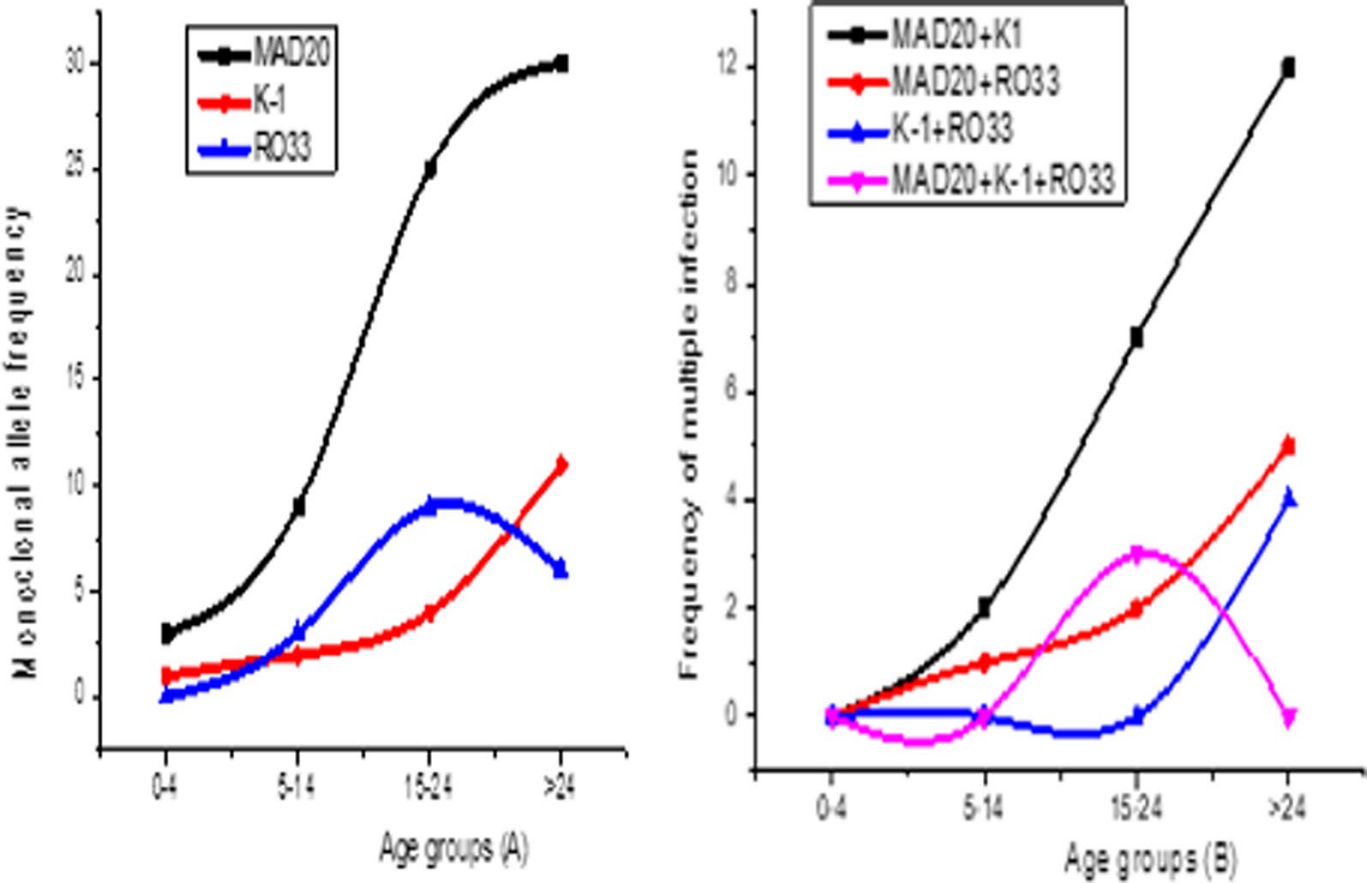
The frequency of monoclonal (**A**) and polyclonal (**B**) allele typing of msp-1 gene across different age groups of malaria patients due to *P. falciparum* in Adama and its surroundings (n = 139)

From all the age groups, 74% of the isolates had monoclonal infections (Fig. 3A). The prevalence of multiple infections slightly increases with age group (Fig. 3B). However, no significant correlation exists between parasite density and multiple infections (Pearson’s correlation = − 0.07, X^2^ = 0.6) and age of the patient with parasite density (Pearson’s correlation = 0.12, X^2^ = 0.6) (Fig. 2). Similarly, there was no significant variation in *msp-1* allelic families with age (X^2^ = 0.5), sex (X^2^ = 0.56), residence (X^2^ = 0.2), travel history (X^2^ = 0.9), educational level (X^2^ = 0.8) and occupation (X^2^ = 0.5) (Table 1).

### Allelic Polymorphism of block 2 region of msp-1 gene and their level of severity, spatial and seasonal features

From the total of 139 successfully genotyped samples by nested PCR; the frequency of *msp-1* allelic families detected in monoclonal isolates were 48%, 13%, 13% for MAD20, K1 & RO33, respectively, and the remaining 24% were diclonal (MAD20 + K-1, MAD20 + RO33, K-1 + RO33) and 2% triclonal (MAD20 + K1 + RO33) infections. From all *P. falciparum msp-1* gene amplified by nested PCR for block 2 region, 19 different alleles were identified of which 8 alleles were MAD20 (160–280 bp), 6 alleles were K-1(100–270 bp), and 5 alleles of RO33 type (100–200 bp). The overall MOI was 1.3, with the expected heterozygosity of 0.39 (Table 2).

**Table 2:** Genetic diversity and genotype multiplicity of *P. falciparum* clinical isolates from Symptomatic uncomplicated malaria patients in Adama and its surroundings (n = 139)

Msp-1 alleles (n = 139)	Frequency %	Allele size (bp)	Number of alleles	Overall MOI	*He*
K-1	18 (12.9)	100–270	6	1.3	0.39
MAD20	67 (48.2)	160–280	8		
RO33	18 (12.9)	100–200	5		
K-1 + MAD20	21 (15.1)				
K-1 + RO33	4 (2.9)				
MAD20 + RO33	8 (5.8)				
K-1 + MAD20 + RO33	3 (2.2)				
Total	139				

Of the total multiclonal infections 29 (80%) were detected during the major malaria season (September to December) and the rest were from the isolates of minor malaria season in the region. No statistically significant variation in the seasonal distribution of polyclonal infection (X^2^ = 0.8) in the study area. Moreover, 33 (92%) patients with polyclonal infection were having no travel history to other malaria endemic places. Thus, there was no statistically significant variation in the distribution of allelic variants in relation to patient’s travel history in the study area (X^2^ = 0.9) (Table 3).

**Table 3:** The relationship between polyclonal infections, its seasonality and travel history of malaria patient due to *P. falciparum* in Adama and its surroundings (n = 139)

Allelic type	Season		Chi-square (X^2^)	Travel history		Chi-square (X^2^)
	Major	Minor		Yes	No	
MAD20 + K-1	16	5	0.8	2	18	0.9
MAD20 + RO33	8	0		1	7	
K-1 + RO33	3	1		0	4	
MAD20 + K-1 + RO33	2	1		0	3	
Total	29	7		3	33	

In this study, of all *P. falciparum* isolates; 83 (60%) were from the urban locality, and the rest were from rural area (Table 4). Allelic variants of *msp-1* did not show significant variation between urban and rural areas; and seasonal variations were not statistically significant (X^2^ = 0.23) and (X^2^ = 0.57), respectively.

**Table 4:** Rural, urban and seasonal variations in the distribution of *P. falciparum* msp-1 block 2 region allelic variants in Adama and its surroundings (n = 139)

Msp-1 block 2 allele types	Number of positive alleles	Rural	Urban	X^2^	Major malaria season	Minor malaria season	X^2^
MAD20	67	25	42	0.23	46	21	0.57
K-1	18	11	7		13	5	
RO33	18	10	8		15	3	
MAD20 + K-1	21	5	16		16	5	
MAD20 + RO33	8	3	5		8	0	
K-1 + RO33	4	3	1		3	1	
MAD20 + K-1 + RO33	3	1	2		2	1	

**Fig. 4:**
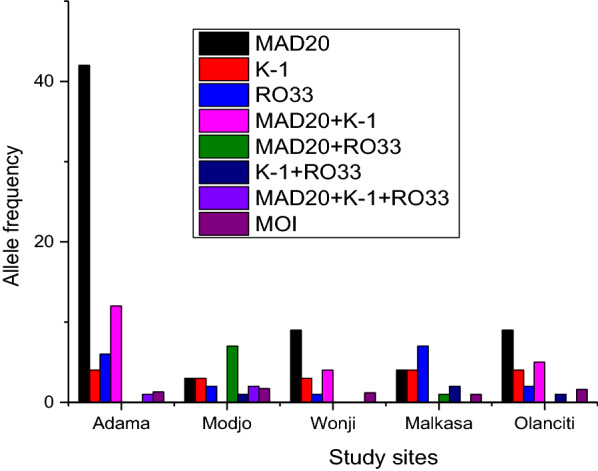
Distribution P. falciparum msp-1 gene allelic families isolated in Adama and its surroundings (n = 139)

Analysis of the spatial feature of *msp-1* allelic variants and MOI from the study sites has shown that 65 (47%), 18 (13%), 17 (12%), 18 (13%), 21(15%) isolates were from Adama, Modjo, Wonji, Malkasa, and Olanciti sites, respectively. The spatial variation of the distribution of *msp-1* allelic variant across sample collection sites was significantly related (P = 0.000) (Fig. 3) showing heterogeneity in their distribution (Fig. 4).

## Discussion

In Ethiopia, even though considerable efforts have been made at national and local levels to control and eventually eliminate malaria, limited molecular data exists on genetic polymorphism of *P. falciparum,* the most predominant and virulent malaria parasite in the region. The present study aimed to assess the genetic polymorphism of *P. falciparum* clinical isolates based on block 2 region *msp-1* genotypes and multiplicity of infection. This is the first study that widely investigated the status of *P. falciparum* genetic diversity from three districts of the study areas in central Ethiopia. Moreover, the study examined the spatial and seasonality of such polymorphism in relation to parasite density and other patient characteristics.

The study revealed that; geometric mean of parasite density was disproportionately high in school age children (SAC) and relatively stable afterwards (Fig. 2). In addition, there was no statistically significant correlation existed between parasite density and age of the patients (Pearson’s correlation = 0.12, P = 0.6). Even though a number of factors may contribute to the fluctuation of parasitaemia level overtime in symptomatic patients, the geometric mean of microscopically detectable parasitaemia levels could be used to explain the finding of this study [24]. The major factor that mainly contributed for higher parasitaemia level in SAC is delayed acquisition of protective immunity during this immunological transition age making this age group more vulnerable to malaria infection than adults [25].

In the present study, multiple infections slightly increased with age group (Fig. 2B), although the variation was not statistically significant (X^2^ = 0.5). This finding is in congruent with the report from Burkina Faso [26] and Tanzania [27], where they explained that episodes of infection in children is commonly for very short duration and the duration of episodes of infection increases with age contributing to the multiple infections. Other reports suggested that multiple infections vary with parasite density, immunity status, the overall prevalence of infection in the population and transmission intensity as reviewed by [28–30]. Other studies have shown an inverse association. Therefore, the relationship between malaria patient age, level of parasitaemia, number of clones of infection, transmission intensity and status of immunity to malaria parasite needs further investigation.

In the present study, there was no significant correlation existed between multiple clone infections of *P. falciparum* with seasonal variation of malaria incidence and travel history of patients (Table 3). In favour of this finding, report from southwestern Ethiopia [31], has shown the absence of correlation or negative correlation between the proportion of multi-clonal infections and parasite prevalence. On the other hand, reports from Indonesia [32], and Papua New Guinea [33], show the presence of positive correlation between the rate of polyclonal infections and annual parasite incidence. The predominance of polyclonarity (92%) in those patients having no travel history depicts real features of malaria epidemiology with respect to the genetic marker of *msp-1* gene in the study area.

In this study, 26% of the isolates having multiple genotype infections. The overall MOI of 1.3 and the expected heterozygosity of 0.39 (Table 2). This finding differs from north western Ethiopia and southwestern Ethiopia reported by Mohammed et al*.* [23] and Abamecha et al*.* [34] with 75% and 80% frequency of multi-clonal infections, and 1.8 MOI with *He* (0.79), 2.0 MOI and *He* (0.43), respectively. This shows that malaria transmission in the study under report exhibits slightly low genetic diversity, compared with northwestern and southwestern Ethiopia. This could be due the locational advantage of central Ethiopia to better health services, differences in local epidemiology, demographic and environmental conditions that might have resulted in observed reduced genetic diversity pattern in Adama and its surroundings. In the present study, from 139 samples 19 different length polymorphism of *msp-1* allelic variant was revealed; 8 MAD20 (160–330 bp), 6 K-1 (100–270) bp, and 5 RO33 (100–220 bp). This shows the level of size polymorphism of *msp-1* alleles in the study area. However, the number of alleles identified may have been under estimated due to a number of limitations like sensitivity of PCR technique used, inability to differentiate minor fragments, the possible existence of similar size fragments and the same size fragment having different amino acid motifs [34, 35].

Size polymorphism of *msp-1* allelic variant identified in the present study is slightly higher than the report from Chewaka district of southwestern Ethiopia [34] and Humera of north-western Ethiopia [6]. This was less diverse than Kolla Shele district of south western part of Ethiopia [23], but more or less similar to reports from Equatorial Guinea [22], and Bobo-Dioulasso in Burkina Faso [36]. The major factor that may account for such variation could be the scope of study sites covered and local malaria transmission patterns might have contributed. Gel- analysis of the present study revealed that 103 out of 139 *msp-1* amplicon (74%) were monoclonal infections, whereas the remaining 36 (26%) was poly-allelic type, with 15% for (MAD20 + K-1), 5.7% for (MAD20 + RO33), 2.8% for (K-1 + RO33), and 2.1% were MAD20 + K-1 + RO33 type. The proportion of monoclonal infection was 48% MAD20, 13% K-1 and 13% RO33 (Table 2). This finding differ from the report from southwestern Ethiopia [23, 34], where they reported that K-1 was the most prevalent allelic family. Similarly, report from Cameroon, Gambia, Nigeria and Gabon has shown that MAD20 allelic variant was the least predominant [37, 38]. On the other hand, in agreement with the present study report from northwestern part of Ethiopia [6], Sudan by [7] and Equatorial Guinea [22] of the three *msp-1* gene allelic families MAD20 was the predominant allelic type. Although the deriving forces for such variation needs further investigation; the difference in micro-ecological factors and the local transmission intensity [39, 40], could play a significant role. Moreover, evolutionary process like genetic drift resulting uneven reproduction of the parasite lineages, types and rate of mutations, inbreeding and the contribution of allelic variants in reproductive success are some of the factors that might have contributed for such variation [41]. In addition, in the present study when the spatial feature of the distribution of *msp-1* gene allelic variant in urban and rural areas (Table 4) was examined, no statistically significant (P = 0.2) variation was revealed. This finding could be taken as an evidence to show similar malaria epidemiology and the possible crossbreeding of the parasite populations between urban and rural settings in the study area, demanding similar intervention endeavours. Similarly in the present study, no statistically significant variation of multi-clonal infection of *msp-1* gene with parasite density (P = 0.6), and seasonality of transmission (P = 0.8). This could be due to the characteristic feature of low transmission settings in such malaria endemic regions [42, 43]. On the other hand, study sites based distribution of allelic variants has shown a highly significant variation (P = 0.000), (Fig. 4). This could be due to the difference in local micro-ecology of the areas, intensity of local transmission pattern, and differences in the age of the study population [36, 44] and the relative potential differences and challenges on the ongoing malaria control and elimination endeavours in those sites.

This study is the first attempt to analyse the most polymorphic gene (*msp-1*) of *P. falciparum* population in the study area. However, further characterization of this gene needs to be designed by increasing the sample size, use of the most powerful techniques, such as microsatellite DNA sequencing and capillary electrophoresis that would provide strong molecular evidence for malaria parasite genetic profile.

## Conclusion

The study revealed that slightly low genetic diversity of *P. falciparum* clinical isolates found in the study area. Moreover, high frequency of MAD20 allelic variant form was detected. The driving force for such selective advantage for this allele under declining malaria prevalence in our study area demand further investigation. Thus, this information will serve as a baseline molecular evidence for further research on areas having similar malaria epidemiology for targeted interventions to make the control and elimination efforts more effective (Additional files 1, 2).

## Supplementary Information

**Additional file 1.** Primer design.

**Additional file 2.** msp-1 allelic fragment size using 50 bp ladder identified by gel electrophoresis.

## Data Availability

All relevant data is included in manuscript, and the datasets analyzed in the study are available from the corresponding author on reasonable request. Additional data uploaded with main document.

## Abbreviations

Bp: Base pair
msp-1: Merozoite surface protein-1
MOI: Multiplicity of infection
PCR: Polymerase chain reaction
SAC: School age children
