# Supplementary material for: Detection of high frequency of MAD20 allelic variants of Plasmodium falciparum merozoite surface protein 1 gene from Adama and its surroundings, Oromia, Ethiopia
Source: Malar J. 2021 Sep 27;20:385. doi: 10.1186/s12936-021-03914-9 (PMC8477549; doi:10.1186/s12936-021-03914-9)
Supplement: Supplementary file 1 — Additional file 1. Primer design. [file 12936_2021_3914_MOESM1_ESM.docx]

| S.N | Primer name | Sequence | Target gene/allele |
| --- | --- | --- | --- |
| 1. | M1-OF | 5’-CTA GAA GCT TTA GAA GAT GCA GTA TTG-3’ | MSP-1 |
|  | M1-OR | 5’ CTT AAA TAG TAT TCT AAT TCA AGT GGA-3’ |  |
| 1.1 | M1-KF | 5’- AAT GAA GAAGAA ATT ACT ACA AAA GGT-3’ | K-1 allele |
|  | M1-KR | 5’- GCT TGC ATC AGC TGG AGG GCT TGC ACC-3’ |  |
| 1.2 | M1-MF | 5’- AAA TGA AGG AAC AAG TGG AAC AGC TGT-3’ | MAD20 allele |
|  | M1-MR | 5’-ATC TGA AGG ATT TGT ACG TCT TGA AT-3’ |  |
| 1.3 | M1-RF | 5-TAA AGG ATG GAG CAA ATA CTC AAG TTG TTG-3’ | RO33 allele |
|  | M1-RR | 5’-CAT CTG AAGGAT TTG CAG CACCTG GAG-3’ |  |

Table . Oligonucleotide sequences used in the study to amplify outer region (N-1), and inner allelic families (N-2) of msp-1 gene
