# Supplementary figures and images for: Detection of high frequency of MAD20 allelic variants of Plasmodium falciparum merozoite surface protein 1 gene from Adama and its surroundings, Oromia, Ethiopia
Source: Malar J. 2021 Sep 27;20:385. doi: 10.1186/s12936-021-03914-9 (PMC8477549; doi:10.1186/s12936-021-03914-9)

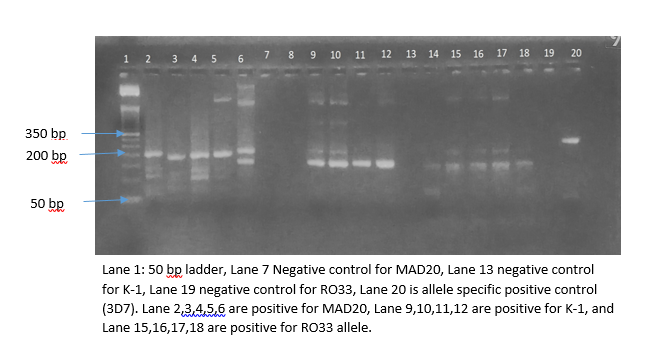

Supplement: Supplementary file 2 — Additional file 2. msp-1 allelic fragment size using 50 bp ladder identified by gel electrophoresis. [file 12936_2021_3914_MOESM2_ESM.docx]
